# Integrated Transcriptomic and Proteomic Analysis of Red Blood Cells from Rainbow Trout Challenged with VHSV Point Towards Novel Immunomodulant Targets

**DOI:** 10.3390/vaccines7030063

**Published:** 2019-07-09

**Authors:** Ivan Nombela, Marina Lopez-Lorigados, Maria Elizabeth Salvador-Mira, Sara Puente-Marin, Veronica Chico, Sergio Ciordia, Maria Carmen Mena, Luis Mercado, Julio Coll, Luis Perez, Maria del Mar Ortega-Villaizan

**Affiliations:** 1 Departamento de Bioquímica y Biología Molecular, Instituto de Biología Molecular y Celular (IBMC) and Instituto de Investigación, Desarrollo e Innovación en Biotecnología Sanitaria de Elche (IDiBE), Universidad Miguel Hernández (UMH), 03202 Elche, Spain; 2 Unidad de Proteómica, Centro Nacional de Biotecnología (CNB-CSIC), 28049 Madrid, Spain; 3 Instituto de Biología, Pontificia Universidad Católica de Valparaíso (PUCV), Valparaíso 2373223, Chile; 4 Departamento de Biotecnología, Instituto Nacional de Investigación y Tecnología Agraria y Alimentaria (INIA), 28040 Madrid, Spain

**Keywords:** erythrocytes, red blood cells, transcriptome, proteome, interferon, complement, VHSV, rhabdoviruses, Mx, IFIT5, β-defensin 1, antigen presentation

## Abstract

Teleost red blood cells (RBCs) are nucleated and therefore can propagate cellular responses to exogenous stimuli. RBCs can mount an immune response against a variety of fish viruses, including the viral septicemia hemorrhagic virus (VHSV), which is one of the most prevalent fish viruses resulting in aquaculture losses. In this work, RBCs from blood and head kidney samples of rainbow trout challenged with VHSV were analyzed via transcriptomic and proteomic analyses. We detected an overrepresentation of differentially expressed genes (DEGs) related to the type I interferon response and signaling in RBCs from the head kidney and related to complement activation in RBCs from blood. Antigen processing and presentation of peptide antigen was overrepresented in RBCs from both tissues. DEGs shared by both tissues showed an opposite expression profile. In summary, this work has demonstrated that teleost RBCs can modulate the immune response during an in vivo viral infection, thus implicating RBCs as cell targets for the development of novel immunomodulants.

## 1. Introduction

Teleost red blood cells (RBCs) have garnered interest in recent years due to the fact that, in contrast to mammalian RBCs, they are nucleated and possess organelles within the cytoplasm [1] and contain the intracellular machinery necessary to develop a response to pathogens [2].

Teleost nucleated RBCs have been recently defined as immune cell mediators of the antiviral response [2,3]. Several immune functions have been associated with RBCs, such as pattern-recognition receptor expression [2,4,5,6], interferon signaling pathway responses [5,6,7,8,9,10], antigen presentation via major histocompatibility complex (MHC) class I (MHCI) or class II (MHCII) [5,7,9,11,12], inflammatory cytokines and chemokines [5,7,9,13], and even the expression of immunoglobulin M in a differentiated state [14].

Different viral pathogens are known to infect teleost RBCs. Among them, the infectious salmonid anemia virus (ISAV) has been described to be endocytosed by RBCs, cause haemagglutination of RBCs [15], and induce the replication and production of type I interferon in vitro [10]. In an in vivo context, ISAV can adhere to the membranes of RBCs and cause RBCs agglutination [16]. Piscine orthoreovirus (PRV) has been also described to infect RBCs. Previous studies have shown infectivity in Atlantic salmon RBCs under both ex vivo and in vivo conditions [17,18]. Similar to ISAV, PRV can increase the expression of type I interferon and its related genes in Atlantic salmon RBCs [9,17]. Moreover, a transcriptomic analysis of RBCs from PRV-challenged Atlantic salmon showed the upregulation of genes related to antigen presentation via MHCI and interferon-regulated genes with antiviral activity [9].

On the other hand, the infectious pancreatic necrosis virus (IPNV), despite not being infective in rainbow trout RBCs, increased the ex vivo expression of type I interferon and interferon-stimulated genes (e.g., myxovirus resistance protein (Mx)) [6]. Similarly, the viral hemorrhagic septicemia virus (VHSV) infection has been reported to be halted in rainbow trout RBCs in conjunction with an RBC antiviral response [8,13]. Moreover, a recent study has shown that RBCs display antigen-processing mechanisms such as autophagy and proteasome activity, as detected by a transcriptomic, proteomic, and a functional analysis of RBCs exposed ex vivo to VHSV [11]. RBCs have been reported to modulate the expression of MHCI and MHCII, the cluster of differentiation 83 (CD83), and the cluster of differentiation 86 (CD86) molecules when they are exposed to VHSV [11], both ex vivo and in vivo. Separately, rock bream RBCs have been reported to generate a response to challenge with the rock bream iridovirus (RBIV) [19]. This response, which was evaluated via proteomic analysis, was mainly characterized by the upregulation of apoptosis- and MHCI-related pathways and the downregulation of interferon-stimulating gene 15 (ISG15) antiviral mechanisms [19].

The objective of this study was to analyze whether rainbow trout RBCs could modulate an immune response in vivo against VHSV to elucidate if teleost RBCs could act as potential immune modulators in the context of viral infections. To further investigate this, we evaluated the global RBC immune response using transcriptomic analyses of RBCs from peripheral blood (PB-RBCs) and head kidney (HK-RBCs), the major hematopoietic organ in fish [20], sorted in a single cell manner. Similarly, a proteomic analysis of the PB-RBCs of rainbow trout challenged with VHSV was performed. We detected the upregulation of genes in the PB-RBCs of VHSV-challenged rainbow trout in several immune-related overrepresented Gene Ontology (GO) Term categories, including the activation of the complement system, cytoplasmic pattern recognition receptor signaling, and the antigen processing and presentation of exogenous peptide, among others. From the proteomic analysis, we found proteins with known immune functions such as nucleotide-binding oligomerization domain (NOD)-like receptor (NLR) with caspase recruitment domain (CARD) domain-containing 3 (NLRC3), the NLR family pyrin domain-containing 12 (NLRP12) inflammasome, and the interferon-induced protein with tetratricopeptide repeat 5 (IFIT5). On the other hand, the HK-RBCs of VHSV-challenged individuals showed upregulated genes related to the type I interferon and the antigen processing and presentation of endogenous peptide antigen via MHCI pathways. Antiviral effectors stimulated by type I interferon, such as Mx and IFIT5, were highly expressed in PB-RBCs from VHSV-challenged rainbow at both the transcript and protein levels. In summary, RBCs can develop an immune response during an in vivo VHSV infection of rainbow trout, despite these RBCs not being infected by VHSV. Our results implicate RBCs as potential cell targets for the development of novel immunomodulants for aquaculture-farmed species.

## 2. Materials and Methods

### 2.1. Animals

Rainbow trout (*Oncorhynchus mykiss*), male and female, individuals of approximately 5–6 cm (about 2 months after hatching) were obtained from a commercial fish farm (Piszolla S.L., Cimballa Fish Farm, Zaragoza, Spain). Fish were maintained at the University Miguel Hernandez (UMH) facilities in a recirculating, dechlorinated, and progressively microfiltered (up to 0.2 µm pore size) water system and fed once a day with a commercial diet for rainbow trout fingerling (Nutra Sprint, Skretting, Burgos, Spain). Water temperature and pH were constantly monitored to maintain fish at 14 °C and 8.0, respectively. Water carbonate hardness and conductivity measures were also maintained about 6° d and 750 µS, respectively. Fish were acclimatized to laboratory conditions for 2 weeks before experimentation. Individuals were sampled using hand nets.

### 2.2. Rainbow Trout Challenge with VHSV

Fingerling rainbow trout individuals were challenged by an intramuscular injection of a 50 µL Roswell Park Memorial Institute (RPMI)-1640 medium (Dutch modification) (Gibco, Thermo Fischer Scientific Inc., Carlsbad, CA, USA) supplemented with 2% fetal bovine serum (FBS) (Cultek, Madrid, Spain), 1 mM of pyruvate (Gibco), 2 mM of L-glutamine (Gibco), 50 µg/mL of gentamicin (Gibco), 2 µg/mL of fungizone (Gibco), and 100 U/mL of penicillin/streptomycin (Sigma-Aldrich, Madrid, Spain) with VHSV (10^8^ tissue culture infectious dose 50% (TCID_50_)/mL). As a negative infection control, individuals were injected with 50 µL of sterile RPMI 2% FBS. Over the course of the challenge, individuals were maintained at 14 °C for the number of days indicated in each assay. The viral hemorrhagic septicemia virus (VHSV-07.71) was purchased from the American Type Culture Collection (ATCC, VR-1388) and propagated in fathead minnow epithelioma papulosum cyprini (EPC) cells [21] at 14 °C, as previously reported [22].

### 2.3. Blood Extraction and Staining

Rainbow trout were sacrificed by overexposure to tricaine methanesulfonate (Sigma-Aldrich) at 300 mg/L. Peripheral blood was sampled from the caudal vein using insulin syringes (NIPRO Bridgewater, NJ, USA). Approximately 100 µL of blood were diluted in RPMI 10% FBS. Blood cells were dyed using SYTO RNASelect (Thermo Fischer Scientific Inc.) diluted 1/1000 in RPMI 10% FBS for 20 min at room temperature. The diluted SYTO RNASelect was removed by centrifugation of the cell suspension at 1600 rpm, and cells were resuspended in RPMI for cell sorting.

### 2.4. Cell Sorting

**Figure 1:**
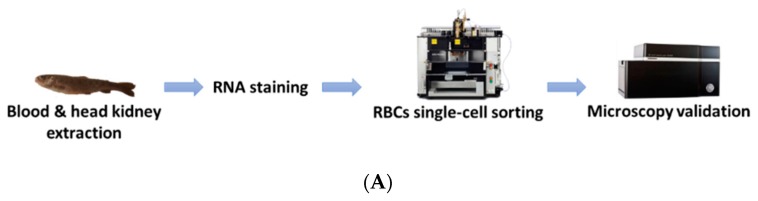

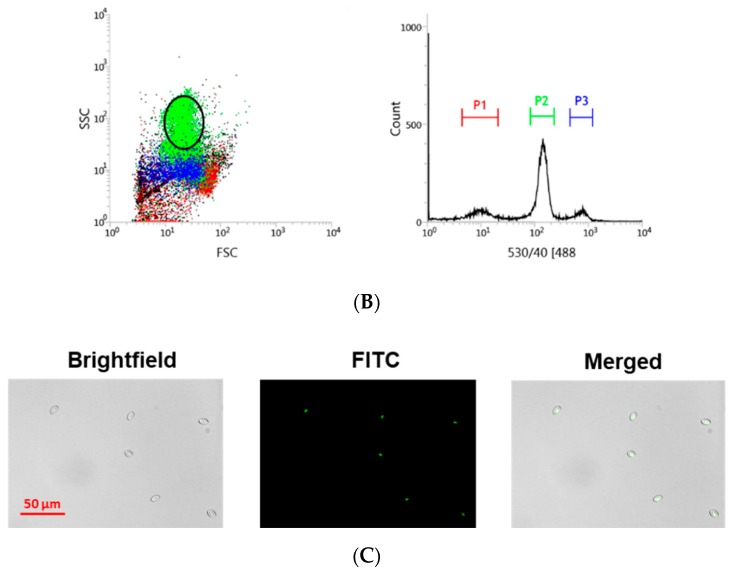
Overview of the single-cell sorting methodology. (**A**) Workflow of single-cell sorting using the BD FACSJazz cell sorter. (**B**) Double gating population selection for RBCs sorting based on: (i) Fluorescence intensity, where the P2 (green) population corresponds to RBCs, and a (ii) forward-scattering (FSC)/side-scattering (SSC) dot plot, where the selected circled population corresponds to RBCs. (**C**) Representative bright-field and fluorescein (FITC) microscopy images of single-cell sorted RBCs from the head kidney (HK), taken with 10× magnification.

RBCs from head kidney and peripheral blood were sorted using the BD FACSJazz cell sorter (BD Biosciences, Madrid, Spain). The single-cell sorting workflow is represented in Figure 1A. Samples were previously labeled with SYTO RNASelect. HK-RBCs (10^2^ cells per individual) were sorted from the previously selected population (circle in Figure 1B) in a forward-scattering (FSC)/side-scattering (SSC) dot plot, which is part of the P2 population in the fluorescence histogram, using a 1.0 drop single-cell mask in the BD FACSJazz software to maximize purity. PB-RBCs (10^6^ cells per individual) were sorted using a 2.0 drop enrich mask to maximize yield. Sample purity was confirmed by optical microscopy using the IN Cell Analyzer 6000 cell imaging system (GE Healthcare, Little Chalfont, UK). A representative image of the RBCs that were single-cell sorted from the HK is shown in Figure 1C.

### 2.5. Transcriptome Analysis

RBCs from 16 individuals were grouped into 2 pools of 8 individuals for each condition (mock-and VHSV-challenged, at 2 days post-challenge (dpc)). RBCs were preserved in a 1/10 dilution of 9.5 μL of a 10× lysis buffer (Clontech, Takara Bio, Mountain View, CA, USA) and 0.5 µL of a RNase inhibitor (Invitrogen, ThermoFisher Scientific Inc.) at −80 °C until complementary DNA (cDNA) library construction. Then, cDNA was produced directly from pooled lysed cells using a SMART-Seq v4 Ultra Low Input RNA Kit (Clontech, Takara Bio). Sequence reads are available at Sequence Read Archive – National Center for Biotechnology Information (SRA-NCBI) accession SRP133501. RNA-Seq library preparation and sequencing were carried out by STABVida Lda. (Caparica, Portugal) as previously described [12].

### 2.6. Proteome Analysis

RBCs were Ficoll-purified, as previously described [13], from control and VHSV-challenged individuals, as described above. At 2 dpc, VHSV-challenged (*n* = 16) and mock (*n* = 16) RBCs (8 × 10^6^ cells per fish) were pelletized by centrifugation (1600 rpm), and the supernatant was removed. Cells were counted using a TC10 Automated Cell Counter (BioRad, Irvine, CA, USA). The cell pellet was washed 3 times with phosphate-buffered saline (PBS), and then it was digested, cleaned-up/desalted, and pooled into 2 pools of 8 individuals for each condition (mock-and VHSV-challenged). Then, the samples were subjected to liquid chromatography and mass spectrometry analysis (LC-MS) as previously described [12]. Log_2_ peptide ratios followed a normal distribution that was fitted using least squares regression. Mean and standard deviation values derived from the Gaussian fit were used to estimate *p* values and false discovery rates (FDR) at a quantitative level. The confidence interval for protein identification was set to <95% (*p*-value < 0.05), and only peptides with an individual ion score above the 1% FDR threshold were considered correctly identified. Only proteins with ≥2 peptide spectrum matches (PSMs) were considered in the quantitation.

### 2.7. Pathway Enrichment Analysis

For transcriptomic and proteomic analyses, pathway enrichment analysis was performed for differentially expressed genes (DEGs) and differentially expressed proteins (DEPs) using ClueGO v2.3.5 [23], CluePedia v1.5.3 [24], and Cytoscape v3.7.0 [25]. The GO Immune System Process and GO Biological Process databases (updated on 23 February 2017) were used. Genes and proteins selected for functional pathway enrichment analysis had a FDR ≤ 0.05. Genes and proteins symbols were identified by sequence homology with *Homo sapiens* using Blast2GO version 4.1.9 (BioBam, Valencia, Spain) [26]. Genes and proteins only identified in fish are indicated in cursive.

### 2.8. RNA Isolation and cDNA Synthesis

The E.Z.N.A. Total RNA Kit (Omega Bio-Tek Inc., Norcross, GA, USA) was used for total RNA extraction in accordance with the manufacturer’s instructions. To eliminate possible residual genomic DNA, a DNAse treatment of the sample was done using TURBO DNase (Ambion, Thermo Fischer Scientific Inc.) following the manufacturer’s instructions. RNA quantification was done with a NanoDrop Spectrophotometer (Nanodrop Technologies, Wilmington, DE, USA).

cDNA was synthesized from RNA using M-MLV reverse transcriptase (Invitrogen, Thermo Fischer Scientific Inc.) as previously described [27]. cDNA was stored at −20 °C until used.

### 2.9. Quantitative PCR

Quantitative reverse transcription PCR (RT-qPCR) was performed in 20 μL reactions using 24 ng of cDNA, 10 μL of TaqMan universal PCR master mix (Thermo Fischer Scientific), and a 900 nM final concentration of each primer (300 nM for NVHSV gene) using the CFX96 System (BioRad). Cycling conditions were 50 °C for 2 min, 95 °C for 10 min, and 40 cycles of 95 °C for 15 sec and 60 °C for 1 min. Gene expression was analyzed by the 2^−ΔCt^ method [28]. The *ef1a* gene was used as an endogenous control. Primer sequences are listed in Table 1.

### 2.10. Antibodies

The rabbit polyclonal antibodies against rainbow trout β-defensin 1 (BD1) [13] and Mx3 [31] were produced in the laboratory of Professor Amparo Estepa. The mouse polyclonal antibody against rainbow trout IFIT5 was produced in the laboratory of Professor Luis Mercado [8]. The rabbit polyclonal antibody against human α-actin (Sigma-Aldrich, Cat. #2066) was used for Western blotting as a loading control.

### 2.11. Western Blotting

RBCs pellets were resuspended at a concentration of 10^8^ RBCs/mL in a PBS buffer with a protease inhibitor cocktail (Sigma-Aldrich). Cells were lysed by freezing and thawing samples 3 times. Cell debris was eliminated by centrifugation at 12,000 rpm for 10 min. Then, 50 µg of each sample were loaded on a 12% (for Mx3 and IFIT5) and 18% (for BD1) polyacrylamide gel under reducing conditions. Electrophoresis was performed at 150 V for 90 min. Proteins in the gel were transferred to 0.4 µm pore size nitrocellulose membranes (BioRad) for 120 min at 100 V in a transfer buffer (2.5 mM Tris, 9 mM glycine, 20% methanol). Membranes were then blocked in PBS containing 5% dry milk and 0.1% Tween-20 (PMT—(PBS milk tween) buffer), and they were incubated with primary antibodies in a PMT buffer (5% milk for Mx3 and IFIT5 antibodies and 0.5% for BD1 antibody) overnight at 4 °C. Membranes were then washed 3 times for 10 min each with a PBS 0.1%Tween-20 buffer and then incubated with secondary antibody GAR-Po (Sigma-Aldrich) or GAM-Po (Sigma-Aldrich) in a PMT buffer (5% milk for Mx3 and IFIT5 antibodies and 0.5% for BD1 antibody) for 60 min. Membranes were then washed 3 times with PBS 0.2% Tween-20. Peroxidase activity was detected using enhanced chemiluminiscence (ECL) reagents (Amersham Biosciences, Buckinghamshire, UK) and revealed using a ChemiDoc XRS+ system (BioRad). Protein band images were processed using Image Lab software v6.0.1 (BioRad).

### 2.12. Ethics Statement

Experimental procedures on experimental animals were reviewed and approved by the Animal Welfare Body and the Research Ethics Committee at the UMH (approval number 2014.205.E.OEP; 2016.221.E.OEP) and by the competent authority of the Regional Ministry of Presidency and Agriculture, Fisheries, Food and Water supply (approval number 2014/VSC/PEA/00205). All procedures were carried out in accordance with the Spanish Royal Decree RD 53/2013 and EU Directive 2010/63/EU for the protection of animals used for research experimentation and other scientific purposes.

### 2.13. Software and Statistics

All figures and graphics show the mean and standard deviation of the data. *p* values associated with each graphic are represented in the figure legends: * *p*-value < 0.05; ** *p*-value < 0.01. Graphpad Prism 6 (www.graphpad.com) (Graphpad Software Inc., San Diego, CA, USA) was used to prepare graphs and perform statistical calculations. ImageJ software (version 1.51, National Institutes of Health, Bethesda, MD, USA) was used for Western blot band densitometry. Venny 2.1 (www.bioinfogp.cnb.csic.es/tools/venny) was used for Venn diagram construction. The clustering of gene expression was performed using ClustVis (https://biit.cs.ut.ee/clustvis/) [32].

## 3. Results

### 3.1. Transcriptomic Analysis of PB-RBCs and HK-RBCs from VHSV-Challenged Rainbow Trout Revealed the Upregulation of Genes Related to the Complement System and the Interferon Pathway, Respectively

**Figure 2:**
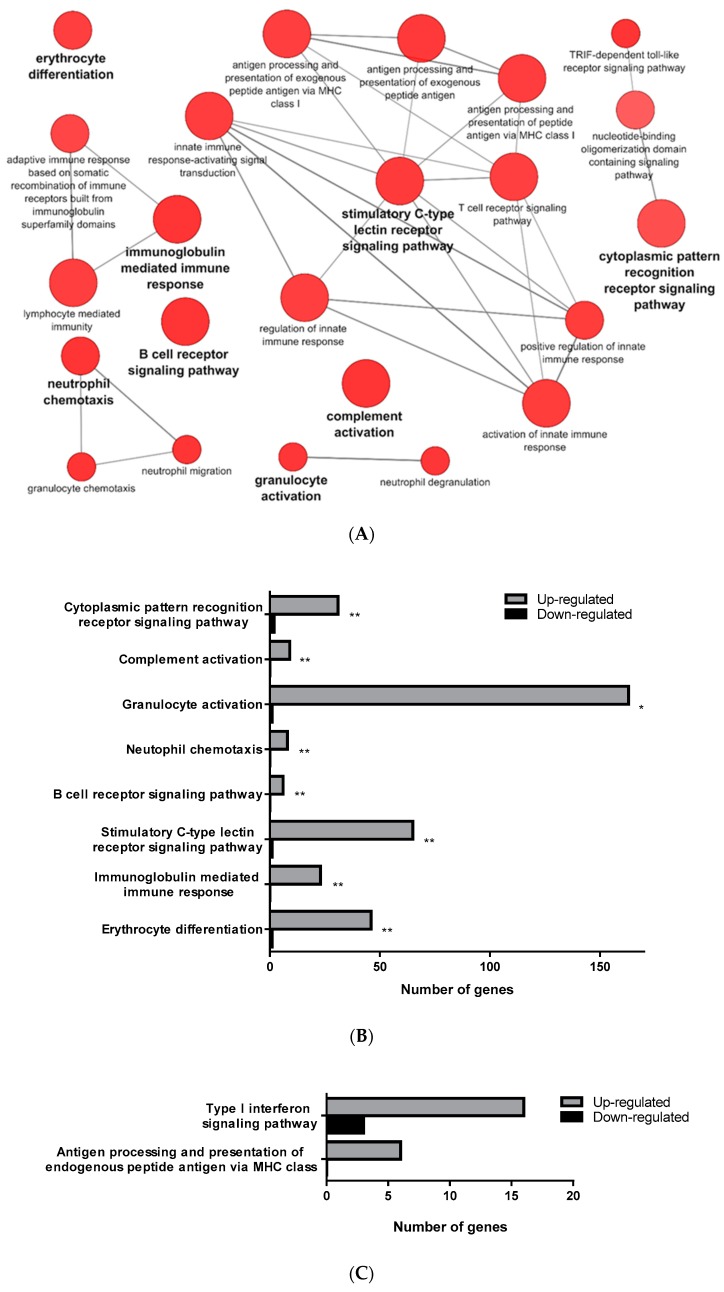

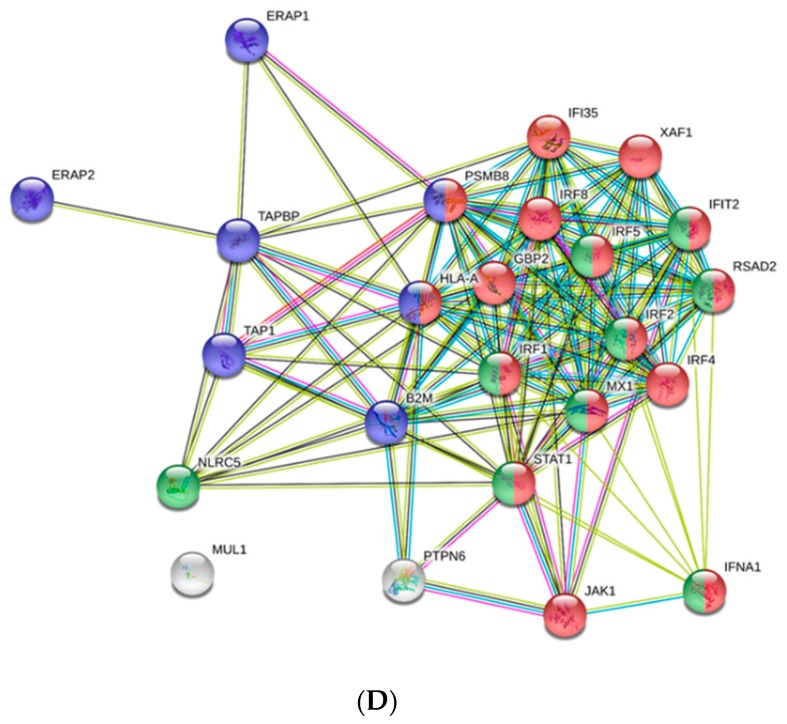
GO Immune System Process categories overrepresented in the transcriptomic analysis of PB-RBCs and head kidney HK-RBCs from VHSV-challenged rainbow trout. (**A**) Pathway enrichment network analysis under the GO Immune System Process database from PB-RBCs of VHSV-challenged rainbow trout. Pathway enrichment analysis was performed selecting GO Immune System Process terms with *p*-value < 0.05, GO Term fusion, and GO Tree interval of 3–8. Red indicates upregulated pathway. (**B**) The number of upregulated and downregulated genes in each represented GO Immune System Process category from VHSV-challenged rainbow trout PB-RBCs. Asterisks denote GO term significance. (**C**) Number of upregulated and downregulated genes in each overrepresented GO Immune System Process category from VHSV-challenged rainbow trout HK-RBCs with the following analysis parameters: *p*-value < 0.1, GO Term fusion, and GO Tree interval of 3–8. (**D**) Protein–protein interaction (PPI) networks of DEGs identified in the GO Immune System Process terms of HK-RBCs from VHSV-challenged rainbow trout constructed using STRING software (*p*-value < 10^−16^). Nodes represent proteins, while edges denote the interactions between two proteins. Different line colors represent the types of evidence used in predicting the associations: Gene fusion (red), gene neighborhood (green), co-expression (black), gene co-occurrence (blue), experimentally determined (purple), from curated databases (teal), text-mining (yellow), or protein homology (lilac). Red nodes denote proteins implicated in the type I interferon signaling pathway (GO:0060337), blue nodes denote proteins implicated in antigen processing and presentation (GO:0019882), and green nodes denote proteins implicated in defense response to virus (GO:0051607).

The transcriptomic analysis comparing RBCs from mock- and VHSV-challenged rainbow trout revealed a differential regulation of 4196 (4137 upregulated and 59 downregulated) genes for PB-RBCs and 1578 (841 upregulated and 737 downregulated) for HK-RBCs. A list with all DEGs in PB-RBCs and HK-RBCs is shown in Supplementary Tables S1 and S2, respectively. Using Cytoscape software and the GO Immune System Process database, we identified the following majorly overrepresented pathways: (i) The activation of complement system, (ii) granulocyte activation, (iii) neutrophil chemotaxis, (iv) immunoglobulin-mediated humoral response, (v) erythrocyte differentiation, (vi) B-cell receptor signaling pathway, (vii) stimulatory C-type lectin receptor signaling pathway, and (viii) cytoplasmic pattern recognition receptor signaling (Figure 2A,B) (Supplementary Table S3) in PB-RBCs from VHSV-challenged rainbow trout. The following pathways were also overrepresented to a lesser degree: (i) The antigen processing and presentation of exogenous peptide antigen and (ii) the Toll/interleukin-1 receptor (TIR)-domain-containing adapter-inducing interferon-β (TRIF)-dependent Toll-like receptor signaling pathway. Among the genes related to complement activation, we highlight the following: Complement component 4 binding protein alpha (*c4bpa*), with a log_2_ fold change (FC) of 7.17; the cluster of differentiation 55 (*cd55*), also known as complement decay-accelerating factor, with a log_2_FC of 4.03; and the cluster of differentiation 59 (*cd59*) with a log_2_FC of 8.75. Among the genes related to cytoplasmic pattern recognition receptor signaling, we highlight DExH-box helicase 58 (*dhx58*) (log_2_FC of 8.69), interferon-induced with helicase C domain 1 (*ifih1*) (log_2_FC of 8.20), interleukin 1 receptor associated kinase 1 (*irak1*) (log_2_FC of 5.12), NLR family member X1 (*nlrx1*) (log_2_FC of 3.81), nucleotide-binding oligomerization domain-containing protein 2 (*nod2*) (log_2_FC of 11.54), and mitochondrial antiviral signaling protein (*mavs*) (log_2_FC of 3.32). In addition, we highlight genes related to the TRIF-dependent Toll-like receptor signaling pathway, including TNF receptor-associated factor 3 (*traf3*) (log_2_FC of 4.84), and genes related to antigen presentation, including *cd83* (log_2_FC of 8.19), *mhcI* (log_2_FC of 11.04), and *mhcII* (log_2_FC of 4.93).

On the other hand, HK-RBCs from VHSV-challenged rainbow trout showed a high upregulation of genes related to type I interferon signaling and the antigen processing and presentation of endogenous peptide antigen via MHCI pathways (Figure 2C) (Supplementary Table S4). Among the genes within the type I interferon signaling pathway, we highlight upregulation in the NOD-like receptor family CARD domain-containing 5 (*nlrc5*) with a log_2_FC of 11.79; guanilate binding protein 2 (*gbp2*) with a log_2_FC of 5.76; signal transducing activating factor (*stat1*) with a log_2_FC of 4.45; Mx dynamin like GTPase 1 (*mx1*) with a log_2_FC of 6.76; *ifit5* with a log_2_FC of 4.85; and interferon induced protein 35 (*ifi35*) with a log_2_FC of 5.12. Genes identified in the GO Immune System Process terms from HK-RBCs of VHSV-challenged rainbow trout interacted strongly, as revealed by a protein–protein interaction (PPI) network analysis (Figure 2D). The PPI network also corroborated the overrepresentation of type I interferon signaling pathway and the antigen processing and presentation pathways.

**Figure 3:**
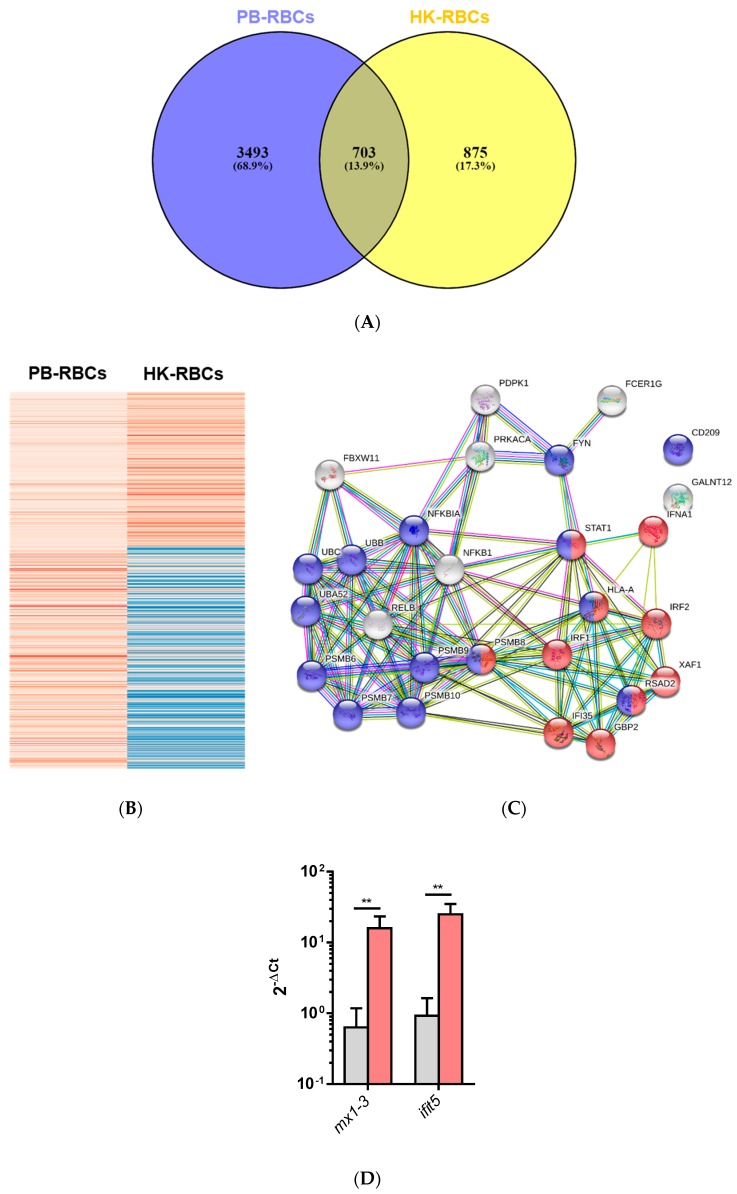
Comparative overview of DEGs shared by HK-PBCs and PB-RBCs. (**A**) Venn diagram of DEGs in RBCs from each tissue. The middle region shows genes expressed in RBCs from both tissues. (**B**) Clustering of gene expression of common DEGs in HK-RBCs and PB-RBCs was performed using ClustVis. The dataset was inserted into matrix category. Parameters for clusterization included no data transformation and no row scaling, and the principal component analysis (PCA) method used was singular value decomposition (SVD) with imputation. Red indicates higher expression and blue represents lower expression. (**C**) PPI networks of the DEGs shared between PB-RBCs and HK-RBCs from VHSV-challenged rainbow trout identified with GO Immune System Process terms and constructed using STRING software (*p*-value < 10^−16^). Nodes represent proteins, while edges denote interaction between two proteins. Different line colors represent the types of evidence used in predicting the associations: Gene fusion (red), gene neighborhood (green), co-expression (black), gene co-occurrence (blue), experimentally determined (purple), from curated databases (teal), text-mining (yellow), or protein homology (lilac). Red nodes denote proteins implicated in the type I interferon signaling pathway (GO:0060337) and blue nodes denote proteins implicated in viral process (GO:0016032). (**D**) Expression of interferon-stimulated genes *mx1-3* and *ifit5* in PB-RBCs from VHSV-challenged rainbow trout (red bars) compared to mock-challenged (gray bars) as control at 2 dpc. Data represent mean ± SD (*n* = 6). A Mann–Whitney test was performed to test statistical significance between PB-RBCs from both groups. Asterisks denote statistical significance. ** *p*-value < 0.01.

**Figure 4:**
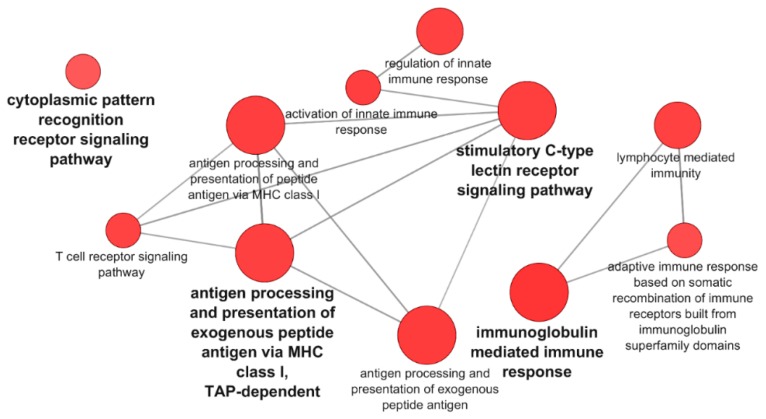
Pathways overrepresented in DEGs exclusive of PB-RBCs from VHSV-challenged rainbow trout. Pathway enrichment analysis was performed using GO Immune System Process terms with *p*-value < 0.05, GO Term fusion, and GO Tree interval of 3–8.

A Venn diagram of the DEGs for PB-RBCs and HK-RBCs (Figure 3A) showed 703 genes common to both samples (13.9% of the total). Supplementary Table S5 shows common and exclusive DEGs for each tissue. Interestingly, DEGs common between PB-RBCs and HK-RBCs that were mainly upregulated in peripheral blood appeared to be downregulated in head kidney and vice versa (Figure 3B). Overrepresented pathways included the type I interferon and stimulatory C-type lectin receptor signaling pathways (Supplementary Table S6). DEGs identified in the GO Immune System Process terms interacted strongly as revealed by a PPI network analysis (Figure 3C). The PPI network also corroborated the overrepresentation of the type I interferon signaling pathway. The gene expression of *mx1-3* and *ifit5* was further analyzed by RT-qPCR in PB-RBCs from VHSV-challenged rainbow trout at 2 dpc (Figure 3D), which showed a statistically significant upregulation. An analysis of DEGs exclusive to PB-RBCs showed overrepresentation of (i) the antigen processing and presentation of exogenous peptide antigen via MHCI, transporter associated with antigen processing (TAP)-dependent; (ii) the stimulatory C-type lectin receptor signaling pathway; (iii) immunoglobulin-mediated immune response; and (iv) the cytoplasmic pattern recognition receptor signaling pathway (Figure 4) (Supplementary Table S7). No significant upregulated or downregulated pathways were detected for DEGs exclusive to HK-RBCs from VHSV-challenged rainbow trout.

### 3.2. Upregulated DEGs from Overrepresented Pathways Were Analyzed by RT-qPCR

**Figure 5:**
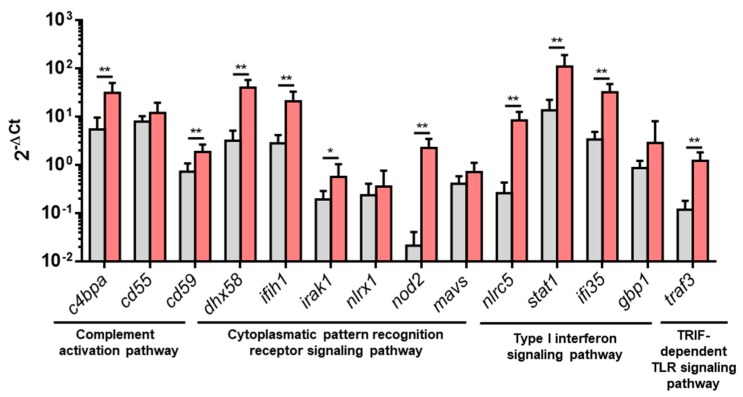
Upregulated DEGs from overrepresented pathways analyzed by RT-qPCR. Gene expression of PB-RBCs from VHSV-challenged rainbow trout (red bars) compared to the control, mock-challenged fish (gray bars) at 2 dpc. Data represent mean ± SD (*n* = 6). A Mann–Whitney test was performed to test statistical significance between PB-RBCs from both groups. Asterisks denote statistical significance. * *p*-value < 0.05; ** *p*-value < 0.01.

An RT-qPCR analysis was performed to further analyze some DEGs from pathways overrepresented in PB-RBCs or HK-RBCs from VHSV-challenged individuals. For the complement activation pathway, we analyzed the expression of *c4bpa*, *cd55*, and *cd59*. For the TRIF-dependent Toll-like receptor signaling pathway, we analyzed *traf3*. For the cytoplasmic pattern recognition receptor signaling pathway, we chose *dhx58*, *ifih1*, *irak1*, *nlrx1*, *nod2*, and *mavs*. From the type I interferon signaling pathway, we analyzed *nlrc5*, *stat1*, guanylate binding protein 1 (*gbp1*) (which is a rainbow trout homolog of human *gbp2*), and *ifi35*. The results showed a statistically significant upregulation in PB-RBCs from VHSV-challenged rainbow trout in all tested genes, except *cd55*, *gbp1*, *mavs*, and *nlrx1,* which were upregulated but not statistically significant (Figure 5). *NVHSV* gene transcripts were barely detectable by RT-qPCR (Cts ranging from 32 to undetected) and transcriptomic analysis. However, the NVHSV protein was not detected in the proteomic analysis.

### 3.3. Proteomic Analysis of PB-RBCs from VHSV-Challenged Rainbow Trout Showed Upregulation of Proteins Involved in the Immune Response

**Figure 6:**
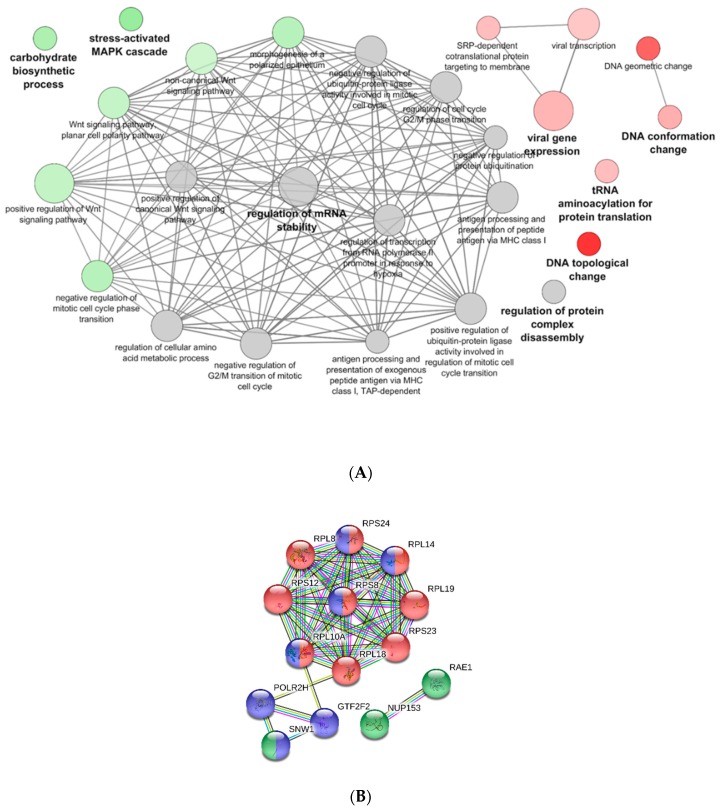
Functional pathway enrichment analysis of DEPs in PB-RBCs from VHSV-challenged individuals. (**A**) A functional pathway enrichment analysis was performed using the GO Immune System Process and GO Biological Process databases with *p*-value < 0.05, GO Term fusion, and GO Tree interval of 3–8. Red indicates upregulated pathways, green indicates downregulated pathways, and gray indicates nonspecific regulation. (**B**) PPI network of proteins associated with the viral transcription GO Term (GO:0019083) using STRING software (*p*-value < 9.99 × 10^−15^). Red nodes denote proteins implicated in translation initiation (GO:0006413). Blue nodes denote proteins implicated in RNA processing (GO:0006396). Green nodes denote proteins implicated in viral process (GO:0016032). Nodes represent proteins, while edges denote interactions between two proteins. Different line colors represent the types of evidence used in predicting the associations: Gene fusion (red), gene neighborhood (green), co-expression (black), gene co-occurrence (blue), experimentally determined (purple), from curated databases (teal), text-mining (yellow), or protein homology (lilac).

PB-RBCs were collected and purified from mock- and VHSV-challenged rainbow trout for proteome analysis. In total, 380 DEPs were detected, 194 of which were upregulated and 186 of which were downregulated. The list of DEPs can be found in Supplementary Table S8. The functional pathway enrichment evaluation of DEPs was performed using the Cytoscape ClueGo platform and the GO Immune System Process database. Overrepresented pathways are listed in Supplementary Table S9. Among these processes, we identified the positive regulation of processes related to viral gene expression and transcription, and categories related to DNA topological change were found to be upregulated as well (Figure 6A). On the other hand, the overrepresented Wnt signaling, carbohydrate biosynthetic process, and stress-activated mitogen-activated protein kinase (MAPK) cascade (Figure 6A) pathways appeared to be downregulated. The regulation of mRNA stability and the antigen processing and presentation of peptide antigen via MHCI pathways appeared to be overrepresented but nonspecifically regulated.

A PPI network using STRING software was performed with the DEPs identified in the viral gene expression and transcription pathways. A high PPI interaction was observed between ribosomal proteins L (RPL) 19, 18, and 8 and ribosomal protein S (RPS) 8 and 12 (Figure 6B). Other proteins, such as eukaryotic translation initiation factor 3 subunit L (EIF3L), RNA polymerase II subunit H (POLR2H), and general transcription factor IIF subunit 2 (GTF2F2), interacted strongly with RPL and RPS proteins (Figure 6B). We would like to highlight the presence and high level of interaction between the nuclear pore complex protein 153 (NUP153) and mRNA export factor RAE1. Among the upregulated DEPs, it is also noteworthy to point out the presence of proteins with known immune functions in viral infections, such as NLRC3 (log_2_FC of 2.79), GBP1 (log_2_FC of 3.71), IFIT5 (log_2_FC of 4.60), IFI35 (log_2_FC of 2.50), radical S-adenosyl methionine (SAM) domain-containing protein 2 (RSAD2) (log_2_FC of 2.08), and GTPase—a very large interferon inducible pseudogene 1 (GVINP1) (log_2_FC of 3.23).

### 3.4. Antiviral Effectors Were Upregulated in VHSV-Challenged Rainbow Trout RBCs

Several antiviral effectors, including cholesterol 25-hydroxylase (*ch25h*), *gvinp1*, *mx*, *ifi35*, *rsad2* (also known as viperin), *ifit5*, interferon-induced transmembrane protein 3 (*ifitm3*), tripartite motif (*trim*) gene family, and sterile alpha motif (SAM) and histidine-aspartate (HD) domain-containing protein 1 (*samhd1*) were identified in PB-RBCs and HK-RBCs from VHSV-challenged rainbow trout and are listed in Table 2 with corresponding log_2_FC. We identified antiviral effectors genes specific to teleost species in PB-RBCs and HK-RBCs, including grass carp reovirus induced gene 2 (*gig2h*), with a log_2_FC of 10.84 and 5.41 in PB-RBCs and HK-RBCs, respectively, and VHSV-induced gene 2 (*vig2*), with a log_2_FC of 6.82 in PB-RBCs.

**Figure 7:**
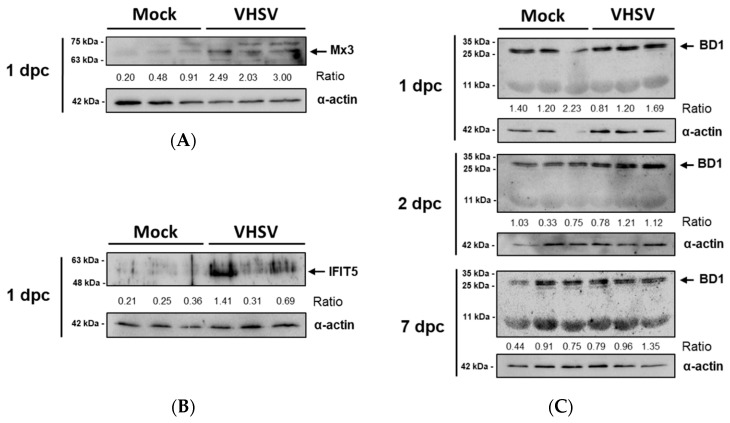
Protein kinetics of interferon-stimulated proteins (**A**) Mx3 (70.8 kDa) [52], and (**B**) IFIT5 (51 kDa) [8], and (**C**) antimicrobial peptide BD1 (monomeric, 7.1 kDa [13], tetrameric, ~28 kDa) in PB-RBCs from mock-and VHSV-challenged rainbow trout at 1, 2, and 7 dpc. Black arrow indicates the band selected for densitometry. Ratio represents expression normalized to α-actin expression.

The expression of the antiviral proteins Mx3 and IFIT5 was analyzed by Western blotting. These proteins were upregulated in PB-RBCs from VHSV-challenged rainbow trout. The Mx3 protein was increased in PB-RBCs from VHSV-challenged individuals at 1 dpc in comparison with mock-challenged fish, as shown by the calculated ratios (Figure 7A). Similarly, IFIT5 was overexpressed at 1 dpc in VHSV-challenged individuals (Figure 7B), following a similar individual expression level trend to Mx3. Via Western blotting, we did not observe protein expression changes for Mx3 and IFIT5 at 2 and 7 dpc (data not shown). We also evaluated the expression of BD1, an antimicrobial peptide known to be induced by interferon [50] which is also considered to be antiviral effector [51]. The BD1 tetrameric form (~28 kDa) expression was higher in PB-RBCs from VHSV-challenged individuals at 2 and 7 dpc, but monomeric BD1 expression (7.1 kDa) did not appear to be altered by a VHSV infection (Figure 7C). The basal expression of these proteins was detected in the mock condition.

## 4. Discussion

Previous reports have shown that RBCs exposed ex vivo to VHSV mainly induced a moderate antiviral response and type I interferon downregulation at early stages after viral exposure and a cellular shut-off at later stages after viral exposure [13]. Autophagy activation, antigen processing, and the upregulation of MHCI, MHCII, CD86, and CD83 antigen-presenting cell markers have been also reported at early time-points after ex vivo VHSV exposure [11]. In the present study, a transcriptomic analysis of PB-RBCs and HK-RBCs from VHSV-challenged individuals showed overrepresentation of processes related to type I interferon signaling, antigen processing, and the presentation of peptide antigen. Cytoplasmic pattern recognition receptor signaling and TRIF-dependent toll like receptor signaling, both related to RNA virus sensing and posterior immune and inflammatory response signaling [53], were detected in PB-RBCs from VHSV-challenged individuals. Among identified cytoplasmic pattern recognition receptors, NOD2 is an intracellular pattern recognition receptor that can interact with mitochondrial antiviral-signaling protein (MAVS) to activate type I interferon signaling in response to RNA viruses [54]. Similarly, NLRX1 has been defined as a regulator of antiviral mitochondrial activity [55]. DHX58, which binds double-stranded RNA (dsRNA), can interact with retinoic acid-inducible gene I (RIG-I) [56]. The helicase activity of DHX58 has been found to have a key role in RIG-I signaling [57]. The melanoma differentiation-associated protein 5 (MDA5), which is encoded by *ifih1* gene, is another receptor with helicase activity, and has been reported to bind viral dsRNA [58]. Among the adaptors, we can find MAVS, which is involved in RIG-I signaling of RNA viruses [59]. TRIF signaling is known to be required for the production of Toll-like receptor (TLR)-mediated type I interferon [60]. The TRIF adaptor has been reported to be a key component of TLR3 double-stranded RNA sensing/signaling cascade [61]. TRIF could therefore be implicated in the signaling cascade of TLRs of RBCs in response to a VHSV infection, finally leading to type I interferon production and secretion. We found an upregulation of NLRP3 in PB-RBCs from VHSV-challenged individuals. NLRP3 has been described as a versatile inflammasome protein that can be activated by a huge number of molecules [62], including RNA viruses [63]. Other pathways exclusively overrepresented in the transcriptomic analysis of PB-RBCs—including stimulatory c-type lectin receptor signaling, B cell receptor signaling, immunoglobulin-mediated immune response, and erythrocyte differentiation—have not been further discussed in this manuscript but are part of our ongoing research.

The PB-RBC transcriptome analysis showed an upregulation of molecules related to complement activation or regulation, such as complement receptor 1 (*cr1*), which binds C3b/C4b complement proteins; complement 4 binding protein alpha (*c4bpa*), which is involved in C4b assembly [64]; and the *cd55* and *cd59* genes, which are involved in the negative regulation of complement activation to prevent RBC lysis [65]. In the 1950s, Nelson described the immunoadherence phenomenon as the binding of antibody-opsonized substrates with C3b, activation of the classical pathway of the complement system, and binding to the surface of RBCs [66,67]. However, the role of rainbow trout RBCs in the immunoadherence/complement response against viral infections is still being studied by our research group.

Molecules implicated in antigen processing and presentation have been previously reported to be upregulated in rainbow trout PB-RBCs exposed to VHSV or from VHSV-challenged individuals [11]. In this study, we also observed the overrepresentation/upregulation of antigen presentation molecules in PB-RBCs and HK-RBCs implicated in the antigen processing and presentation of exogenous and endogenous peptide antigen, respectively. Genes *cd83*, *mhcI*, and *mhcII* appeared highly upregulated in transcriptomic analysis of PB-RBCs from VHSV-challenged rainbow trout. A microarray analysis of RBCs from PRV-challenged Atlantic salmon individuals showed an overrepresentation/upregulation of genes involved in antigen presentation via MHCI, such as MHCI antigens, transporters, and proteasome components [9]. In addition, antigen presentation via MHCI pathway, together with apoptosis processes, were heavily upregulated in RBCs from an RBIV-challenged rock bream [19].

The proteomic analysis mainly identified proteins related to viral transcription. We identified numerous members of the ribosomal S/L proteins that have been previously implicated in viral infections [68] and that closely interact. Viruses may have evolved stimulation mechanisms that synthesize ribosomal proteins to facilitate the translation of their viral components. Moreover, the PPI analysis showed interaction of the RPL/S with EIF3L, POLR2H, and GTF2F2, which have been also implicated in viral replication [69,70,71]. However, we previously described that VHSV replication appeared to be halted in rainbow trout RBCs early after exposure ex vivo [13]. In addition, PB-RBCs from VHSV-challenged rainbow trout only showed low levels of *NVHSV* gene transcripts. Therefore, because viral replication processes appeared to be upregulated, other mechanisms triggered inside RBCs may be interrupting viral replication, such as the autophagy shown in RBCs exposed to VHSV ex vivo [11] or the vast number of antiviral effector ISGs found to be upregulated in RBCs in the present study (Table 2). The integrated ‘omic’ analyses revealed that in response to VHSV, PB-RBCs upregulated genes and proteins that interact with viruses at different stages, such as viral entry or replication, thus acting as antiviral effectors. Such genes/proteins include IFIT5, Mx, GBP1, GVINP1, RSAD2, IFITM3, IFI35, several TRIM proteins, CH25H, and SAMHD1 (supported by the references detailed in Table 2). In addition, we detected the upregulation of *gig2* and *vig2* genes, two molecules known to be induced by RNA virus [72].

The microarray analysis of RBCs from PRV-challenged Atlantic salmon also showed an increased gene expression of antiviral ISGs *mx*, *rsad2*, *gvinp1*, *trim4*, *ifit5*, and *gig2* [9]. In addition, in relation to the type I interferon signaling pathway, the authors observed the upregulation of *tlr3*, *dhx58*, *rig-I*, *stat1*, Janus kinase 1 (*jak1*), *ifn1*, interferon regulatory factor 1 (*irf1*), *irf2*, *irf7*, *irf9*, double stranded RNA-activated protein kinase R (*pkr*), and Z-DNA binding protein kinase (*pkz*) [9]. Our transcriptome results also showed the upregulation of *dhx58*, *stat1*, *jak1*, *ifn1, irf1, irf2*, and *irf7* in RBCs from VHSV-challenged individuals. However, the proteomic sequencing of RBCs from RBIV-challenged rock bream individuals showed the upregulation of STAT1 but the downregulation of TRIM25 and IRF3 proteins [19]. In contrast, these genes were found to be upregulated in this work, in PB-RBCs from VHSV-challenged individuals. Besides, it has been reported that the ISAV infection of Atlantic salmon RBCs ex vivo increased gene expression, by qPCR, of the type I interferon (*ifn1*), *mx*, *isg15*, *stat1*, and *pkz* in haemagglutinated and highly infected RBCs [10]. Similarly, rainbow trout RBCs exposed to IPNV ex vivo showed increased gene expression of *tlr3*, *irf7*, *ifn1*, *mx*, and *pkr,* as well as Mx protein upregulation at longer time-points, despite not being infected [6]. Moderate *ifn1* and *mx* downregulation have been described at early stages after VHSV exposure of RBCs ex vivo, where no haemagglutination nor infection was observed [13]. However, the authors also described *ifn1* paracrine crosstalk between RBCs and rainbow trout gonad 2 (RTG-2) or trout spleen stroma (TSS) cell lines.

In relation to interleukin and chemokine signaling, the microarray analysis of RBCs from PRV-challenged Atlantic salmon individuals mainly showed a global decrease in the gene expression of interleukins, chemokines, and their receptors [9]. In contrast, in the present work, we did not detect a clear or remarkable interleukin/chemokine response in HK-RBCs or PB-RBCs from VHSV-challenged individuals.

Separately, Dahle et al. also showed a global downregulation of complement components in the microarray analysis of RBCs from PRV-challenged Atlantic salmon individuals [9], while, in this work, we detected an overrepresentation/upregulation of the activation of complement system process in PB-RBCs from VHSV-challenged individuals.

As a comparative evaluation of the immune response of nucleated RBCs against a viral challenge or exposure, some of the identified processes, like type I interferon signaling, seemed to be shared in response to the above referenced studied viruses. However, some other processes, like activation of complement system, interleukin/chemokine signaling, or apoptosis, appeared to be differentially regulated in the above referenced studies. It is noteworthy to highlight that PRV [17] and ISAV [10] are known to infect Atlantic salmon RBCs, while VHSV [13] and IPNV [6] cannot actively replicate inside rainbow trout RBCs.

The Mx and IFIT5 proteins, as well as the BD1 antimicrobial peptide, are expressed in response to type I interferon [39,50,73]. In the present work, we demonstrated that PB-RBCs from VHSV-challenged rainbow trout increased the levels of these proteins in transcriptomic and proteomic analyses. Mx is a family of GTPases with antiviral activity [39,74]. High basal expression levels of Mx have been found in rainbow trout RBCs [6,13], and it has been suggested that these high basal levels of Mx may have contributed to the halted VHSV infection in RBCs [2,13]. This has been suggested for other immune cell types as well, such as the rainbow trout spleen monocyte/macrophage (RTS11) cell line [75]. Further, it has been reported that the expression of Mx protein is augmented in response to IPNV exposure [6].

IFIT family proteins present motifs that can bind viral components to prevent or inhibit viral replication [73]. Recently, we detected a correlation between high IFIT5 expression levels in rainbow trout RBCs and a decline in VHSV replication early after VHSV exposure. Moreover, *ifit5* gene silencing increased VHSV replication in rainbow trout RBCs [8]. Therefore, these previous results, in relation to IFIT5 protein in RBCs, are corroborated by the present in vivo study.

The BD1 antimicrobial peptide has been previously reported to be induced by type I interferon [50], and ex vivo exposure of rainbow trout RBCs to VHSV increased BD1 expression [13]. Our results identified the increased expression of BD1 bands of ~28 kDa by Western blotting in VHSV-challenged PB-RBCs. This band size corresponds to a BD1 tetramer, as the monomeric protein is 7.1 kDa [13]. In this regard, it has been previously suggested that β-defensins could form an oligomer to exert antimicrobial activities [76].

Apart from their widely known role in innate immunity, type I interferon response have also been reported to activate an adaptive immune response [77]. Mx has been used as adjuvant for induction of a humoral response against the influenza virus [78]. Also, human beta defensins have been implicated in the activation of adaptive immune response through the induction of antigen-specific immunity [79] and chemotaxis for neutrophils [80]. On the other hand, to our knowledge, there is no reference about the participation of IFIT proteins in adaptive immunity processes.

In summary, we show how nucleated RBCs respond to a VHSV infection in vivo by increasing the expression of genes and proteins related to viral RNA sensing, type I interferon response, ISG antiviral effectors, the antigen processing and presentation of peptide antigen, and complement activation. RBCs are the major cell type in the blood, and understanding their contribution to the antiviral response can allow their use in the development of new prophylactic or therapeutic strategies for viral infections of aquacultured species. Moreover, cytokines and chemokines produced by nucleated RBCs could bridge and enhance their innate and adaptive immune responses. Prophylactic or therapeutic strategies based on antigens coupled with stimulatory adjuvant molecules targeted to RBCs membrane receptors—or even designing of recombinant viral vectors that could selectively target RBCs to induce the expression of antigens and/or co-stimulatory molecules—would be a promising start point of novel prophylactic/therapeutic strategies targeted to nucleated RBCs.

## 5. Conclusions

In this study, we have investigated the immune response of RBCs induced by VHSV in vivo, using an integrated transcriptomic and proteomic analysis. Upregulation and overrepresentation of complement activation and type I interferon signaling processes were revealed in PB-RBCs and HK-RBCs from VHSV-challenged rainbow trout, respectively. Antigen processing and presentation processes appeared overrepresented in RBCs from both tissues. Moreover, in response to the virus, RBCs increased the expression of antiviral genes/proteins, and more specifically proteins such as Mx and IFIT5, and the antimicrobial peptide BD1. In conclusion, rainbow trout RBCs demonstrated to mount a potent antiviral immune response against VHSV in vivo, despite these cells not being infected, and are proposed as new targets for the development of novel prophylactic or therapeutic strategies.

## Figures and Tables

**Table 1:** Primer sequences used in quantitative PCR.

Gene	Forward	Reverse	Probe	Reference or Accession Number
*c4bpa*	TGAGAATGGCGTTAGGATTGAA	GTTGCACTTATACGTCACAAAAGACTT	-	XM_021564396.1
*cd55*	CGCTCAAATTAACCTGCAAAAA	GTGCCTTCCTTAAACTCATATGTCAA	-	XM_021609679.1
*cd59*	CGGAGCCACATCCATTGG	TTACTGCATACACCACCACATCACT	-	NM_001124497.1
*dhx58*	GCTCTCCACTTGCGTCAGTACA	GACCCTAAAGGCATCCACCAT	-	XM_021624832.1
*ef1a*	ACCCTCCTCTTGGTCGTTTC	TGATGACACCAACAGCAACA	GCTGTGCGTGACATGAGGCA	[29]
*gbp1*	TGGTTCCGCTCTAGGTTTCTTC	AGCCTAAAACCCAAAAGAGCAA	-	XM_021579826.1
*ifi35*	CTGGTGCCCTGTCAAGTAGAGA	TTCTTGGGCAGGTTGGAAAC	-	XM_021558400.1
*ifih1*	GAGCCCGTCCAAAGTGAAGTT	AGTGAGGTGTTTTCTCTTTGAATGAA	-	NM_001195179.1
*ifit5*	CCCTCAATGACTCTGACAAGCA	CCCTGCCCTCATCTTTCTTCT	CCAGCTTCGGCCTGTTTCTGTTCCA	[7]
*irak1*	CAGACAGACCAACGCTCACAA	GCAGATCGCACCCACATG	-	XM_021567162.1
*mavs*	GAGGGCAGAGTGGAACAAACA	TCAGAGCTGGTAGAAGGAATTGGT	-	NM_001195181.1
*mx1-3*	TGAAGCCCAGGATGAAATGG	TGGCAGGTCGATGAGTGTGA	ACCTCATCAGCCTAGAGATTGGCTCCCC	[30]
*nlrc5*	CTGCTATGTGCCGCCAATT	CCAGTGTAGGCCAAGGATCAC	-	XM_021570046.1
*nlrx1*	CCTGCTTTTTACCTTCCTATTGCT	CACCTCCCCTCCAAAGTTGA	-	XM_021581927.1
*nod2*	GAGAGACAGGAGTTGACGATTCTG	TTGTCTGACTTCTTCGAGATCATCA	-	NM_001201555.1
*NVHSV*	GACTCAACGGGACAGGAATGA	GGGCAATGCCCAAGTTGTT	TGGGTTGTTCACCCAGGCCGC	[27]
*stat1*	GCCGAGAACATCCCTGAGAA	GCTTACTCGCCAACTCCATTG	-	XM_021596980.1
*traf3*	GGGCTTCAGGGACCACTTC	ACCAGCTTGCAGGACTCACA	-	NM_001124615.1

**Table 2:** Summary of interferon-stimulated genes (ISGs)/antiviral effectors orthologues identified in transcriptomic analyses.

Name	Symbol	Log_2_FC PB-RBCs	Log_2_FC HK-RBCs	References ^1^
Cholesterol 25-hydroxylase	*ch25h*	-	8.68	[33]
GTPase, very large interferon inducible pseudogene 1	*gvinp1*	8.54	-	[34]
Interferon induced protein 35	*ifi35*	8.38	5.12	[35]
Interferon induced protein with tetratricopeptide repeats 5	*ifit5*	14.58	4.85	[8,36]
Interferon-induced transmembrane protein 3	*ifitm3*	5.00	-	[37,38]
Interferon-induced GTP-binding protein Mx 1	*mx1*	10.95	6.76	[39]
Radical SAM domain-containing protein 2	*rsad2*	11.61	5.47	[40,41]
SAM and HD domain-containing protein 1	*samhd1*	7.74	-	[42]
Tripartite motif family 16	*trim16*	8.80	3.95	[43]
Tripartite motif family 21	*trim21*	6.65	-	[44]
Tripartite motif family 25	*trim25*	9.76	7.90	[45,46,47]
Tripartite motif family 39	*trim39*	7.01	8.46	[48]
Tripartite motif family 47	*trim47*	3.75	6.15	[49]

^1^ Related to previous reports about the implication of the respective ISGs as antiviral effectors.

## Supplementary Materials

The following are available online at https://www.mdpi.com/2076-393X/7/3/63/s1, Table S1: Complete list of DEGs identified in the transcriptomic analysis of PB-RBCs from mock- and VHSV-challenged rainbow trout. Table S2: Complete list of DEGs identified in the transcriptomic analysis of HK-RBCs from VHSV-challenged rainbow trout. Table S3: List of GO Immune System Process terms identified in the transcriptomic analysis of PB-RBCs from VHSV-challenged rainbow trout using *p* < 0.05, GO Term fusion, and GO Tree interval 3–8. Table S4: List of GO Immune System Process terms identified in the transcriptomic analysis of HK-RBCs from VHSV-challenged rainbow trout using *p* < 0.1, GO Term fusion, and GO Tree interval 3–8. Table S5: Complete list of common DEGs from both PB-RBCs and HK-RBCs identified in the transcriptomic analysis from mock- and VHSV-challenged rainbow trout. The second sheet lists DEGs found exclusively in PB-RBCs identified in the transcriptomic analysis of mock- and VHSV-challenged rainbow trout. Table S6: List of GO Immune System Process terms identified in the transcriptomic analysis of common DEGs from both PB-RBCs and HK-RBCs from VHSV-challenged rainbow trout using *p*-value < 0.05, GO Term fusion and GO Tree interval 3–8. Table S7: List of GO Immune System Process terms identified in the transcriptomic analysis of DEGs found exclusively in PB-RBCs from VHSV-challenged rainbow trout using *p* < 0.05, GO Term fusion, and GO Tree interval 3–8. Table S8: Complete list of DEPs identified in PB-RBCs from VHSV-challenged rainbow trout. Listed proteins represent those with *p*-value < 0.05, >2 PSMs, and a fold-change greater or less than 1.5. Table S9: List of GO Immune System Process and GO Biological Process terms identified in the proteomic analysis of PB-RBCs from VHSV-challenged rainbow trout using *p*-value < 0.05, GO Term fusion, and GO Tree interval 3–8.
